# 3D nuclear organization of telomeres in the Hodgkin cell lines U-HO1 and U-HO1-PTPN1: PTPN1 expression prevents the formation of very short telomeres including "t-stumps"

**DOI:** 10.1186/1471-2121-11-99

**Published:** 2010-12-14

**Authors:** Hans Knecht, Silke Brüderlein, Silke Wegener, Daniel Lichtensztejn, Zelda Lichtensztejn, Bruno Lemieux, Peter Möller, Sabine Mai

**Affiliations:** 1 Division d'Hématologie, CHUS, Université de Sherbrooke, Sherbrooke, Québec, Canada; 2 Institute of Pathology, University of Ulm, Ulm, Germany; 3 St. Elisabeth Krankenhaus, Köln, Germany; 4 Manitoba Institute of Cellular Biology, University of Manitoba, Winnipeg, Manitoba, Canada

## Abstract

**Background:**

In cancer cells the three-dimensional (3D) telomere organization of interphase nuclei into a telomeric disk is heavily distorted and aggregates are found. In Hodgkin's lymphoma quantitative FISH (3D Q-FISH) reveals a major impact of nuclear telomere dynamics during the transition form mononuclear Hodgkin (H) to diagnostic multinuclear Reed-Sternberg (RS) cells. *In vitro *and *in vivo *formation of RS-cells is associated with the increase of very short telomeres including "t-stumps", telomere loss, telomeric aggregate formation and the generation of "ghost nuclei".

**Results:**

Here we analyze the 3D telomere dynamics by Q-FISH in the novel Hodgkin cell line U-HO1 and its non-receptor protein-tyrosine phosphatase N1 (PTPN1) stable transfectant U-HO1-PTPN1, derived from a primary refractory Hodgkin's lymphoma. Both cell lines show equally high telomerase activity but U-HO1-PTPN differs from U-HO1 by a three times longer doubling time, low STAT5A expression, accumulation of RS-cells (p < 0.0001) and a fourfold increased number of apoptotic cells.

As expected, multinuclear U-HO1-RS-cells and multinuclear U-HO1-PTPN1-RS-cells differ from their mononuclear H-precursors by their nuclear volume (p < 0.0001), the number of telomeres (p < 0.0001) and the increase in telomere aggregates (p < 0.003). Surprisingly, U-HO1-RS cells differ from U-HO1-PTPN1-RS-cells by a highly significant increase of very short telomeres including "t-stumps" (p < 0.0001).

**Conclusion:**

Abundant RS-cells without additional very short telomeres including "t-stumps", high rate of apoptosis, but low STAT5A expression, are hallmarks of the U-HO1-PTPN1 cell line. These characteristics are independent of telomerase activity. Thus, PTPN1 induced dephosphorylation of STAT5 with consecutive lack of Akt/PKB activation and cellular arrest in G_2, _promoting induction of apoptosis, appears as a possible pathogenetic mechanism deserving further experimental investigation.

## Background

The bi- or multinuclear Reed-Sternberg cells (RS-cells), the diagnostic cells of Hodgkin's lymphoma (HL), are derived from their mononuclear precursors, the Hodgkin cells (H-cell) through endoreplication and have a limited capacity to divide further [1-3]. RS-cells appear to be true end-stage tumour cells and their number of nuclei correlates closely with the 3D organization of telomeres [4]. Using a recently developed three-dimensional quantitative fluorescent in situ hybridization technique for telomere (3D telomere Q-FISH) [5] we showed *in vitro *and in diagnostic biopsies that further nuclear division becomes likely impossible because of sustained telomere shortening, loss, aggregation and formation of telomere- and DNA-poor "ghost" nuclei [6]. This process is identified in both, classical EBV-negative and EBV-positive HL [6].

The recently established Hodgkin cell line U-HO1, derived from a patient with primary refractory HL of nodular sclerosis subtype, is EBV negative, expresses CD15 together with CD30 and has a clonal non-functional VDJ-heavy gene rearrangement (Mader et al., 2007). U-HO1 expresses a truncated and non functional form of the non-receptor protein-tyrosine phosphatase PTPN1, has a doubling time of about 4 days under standard culture conditions and forms about 4% of typical RS-cells in suspension [7,8]. Stable expression of PTPN1 in U-HO1 (U-HO1-PTPN1) results in very slow proliferation, substantially increased (about 4 x) RS-cell formation and higher levels of apoptosis [8].

PTPN1 (also called PTP1B) specifically deactivates phosphorylated STAT5A and STAT5B [9] which regulates self-renewal capacity and differentiation of memory B-cells [10]. Phosphorylated STAT5A is highly expressed in all HL-cell lines analyzed so far [11], and its expression is essential for morphogenesis of RS-cells [12]. PTPN1^-/- ^mice show accumulation of large B-cells in bone marrow and lymph nodes [13] as well as increased development of inflammatory macrophages [14]. Moreover, double knock out p53^-/- ^PTPN1^-/- ^mice rapidly develop B-cell lymphomas [13]. These findings are consistent with a significant influence of PTPN1 in B-cell lymphomagenesis on an inflammatory background as present in HL [15].

In order to analyze the 3D nuclear telomere dynamics associated with the transition from H- to RS-cells and to clarify the functional role of PTPN1 expression in this process, we analyzed by 3D telomere Q-FISH both, mononuclear H-cells and multinuclear RS-cells of the U-HO1 and the U-HO1-PTPN1 cell lines, respectively.

## Results

### Growth characteristics

**Figure 1:**
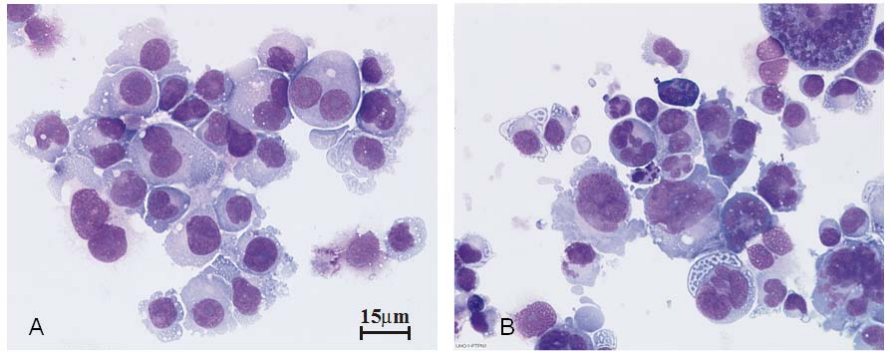
**PTPN1 expression promotes multinuclearity**. Multinuclear RS-cells in U-HO1-mock (A) and U-HO1-PTPN1 (B). May-Grünwald-Giemsa staining.

**Figure 2:**
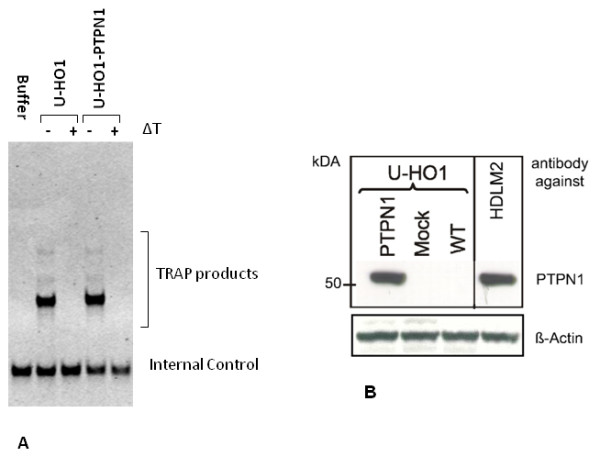
**Telomerase activity and stable PTPN1 expression**. A. TRAP assay shows equal telomerase activity in the HD-cell lines U-HO1 and U-HO1-PTPN. Internal control of each sample after heat denaturation (ΔT +) demonstrates specificity. B. A strong specific band near 50 kDA is observed in U-HO1-PTPN1 and in the positive control, the HD-cell line HDLM-2, whereas no signal is detected U-HO1 and U-HO1-mock.

**Figure 3:**
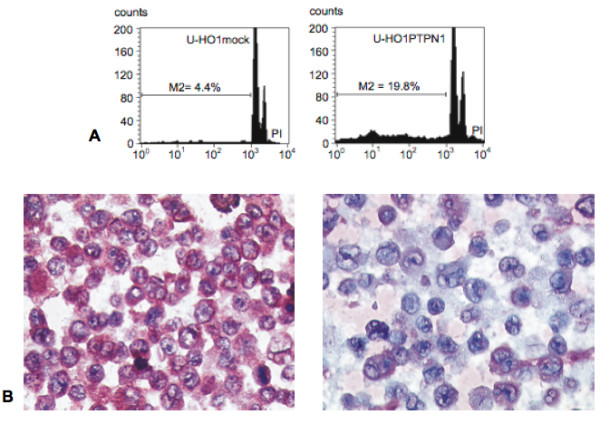
**High apoptosis rate but low STAT5A expression in U-HO1-PTPN1**. A. Apoptotic cells (M2), on the left to the G1 peak on the x-axis, are about 4 times more frequent in U-HO1-PTPN1. B. Abundant phosphorylated STAT5A is present in the cytoplasm of U-HO1 mononuclear H- and multinuclear RS-cells (left panel) but is barely present in U-HO1-PTPN1 (right panel). Note numerous bi- or multinucleated RS-cells in U-HO1-PTPN1 but not in U-HO1.

The HL cell lines U-HO1 had a doubling time of about 3-4 days, and U-HO1-mock about 7 days, whereas U-HO1-PTPN1 grew much slower with a doubling time of about 14 days. In steady state culture, the number of at least bi-nucleated RS-cells was about 4%-5% in both, U-HO1 and U-HO1-mock, but significantly higher (18-22%; p < 0.0001) in U-HO1-PTPN1 (Figure 1A, B). Both cell lines, U-HO1 and U-HO1-PTPN1 had equally high telomerase activity and in U-HO1-PTPN1 stable expression of the specific protein-tyrosin-phosphatase was confirmed by Western blotting (Figure 2A, B). Stable expression of PTPN1 was associated with a high apoptosis rate and had a significant impact on presence of phosphorylated STAT5, which was high in U-HO1 but nearly absent in U-HO1-PTPN1 (Figure 3A, B).

### 3D nuclear organization of telomeres in Hodgkin cells of U-HO1 and U-HO1-PTPN1

**Figure 4:**
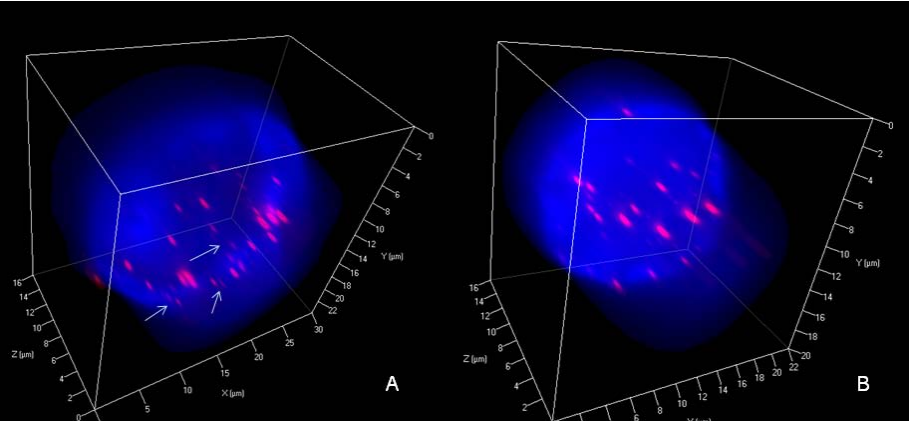
**3D nuclear staining of telomeres and total DNA of H-cells**. A. Mononuclear U-HO1 H-cell (blue) shows few very short (arrows) but mainly short and mid-sized telomeres (red) analogous to those identified in mononuclear U-HO1-PTPN1 H-cells (B).

Mononuclear H-cells of U-HO1 and U-HO1-PTPN1 showed largely identical 3D telomere characteristics (Figure 4 and Table 1). The only difference was a small increase (p = 0.0407) of very short telomeres including so-called "t-stumps" (Xu and Blackburn, 2007), occurring in U-HO1 H-cells (Figure 4 and Table 1).

**Table 1:** 3D telomere characteristics of Hodgkin and Reed-Sternberg cells in U-HO1 and U-HO1-PTPN1

Cell type	Parameter	H-HO1	H-HO1-PTPN1	p-value
**Hodgkin**	Total nuclear volume	4121 ± 1350 μm^3^	3330 ± 844 μm^3^	ns
	Telomeres/cell (n) (Total telomere volume)	53.8 ± 28.0	56.2 ± 19.9	ns
	Telomeres/1000 μm^3^ of nuclear volume (n)	14.0 ± 8.4	17.2 ± 6.1	ns
	Telomere aggregates/cell (n)	6.0 ± 4.0	6.8 ± 4.2	ns
	Cells with ≥ 20 telomere aggregates (%)	2.9	2.8	ns
	Very short telomeres (< 5000 units; %)	21.2	18.3	0.0407
				
**Reed-Sternberg**	Total nuclear volume	15301 ± 4641 μm^3^	14839 ± 6722 μm^3^	ns
	Telomeres/cell (n) (Total telomere volume)	123.0 ± 75.3	105.7 ± 62.7	ns
	Telomeres/1000 μm^3^ of nuclear volume (n)	13.7 ± 30.8	7.8 ± 4.0	ns
	Telomere aggregates/cell (n)	13.8 ± 11.7	12.9 ± 10.5	ns
	Cells with ≥ 20 telomere aggregates (%)	17.2	13.8	ns
	Very short telomeres (< 5000 units; %)	23.4	13.8	< 0.0001

ns: p > 0.05

### 3D nuclear organization of telomeres in Reed-Sternberg cells of U-HO1 and U-HO1-PTPN1

**Figure 5:**
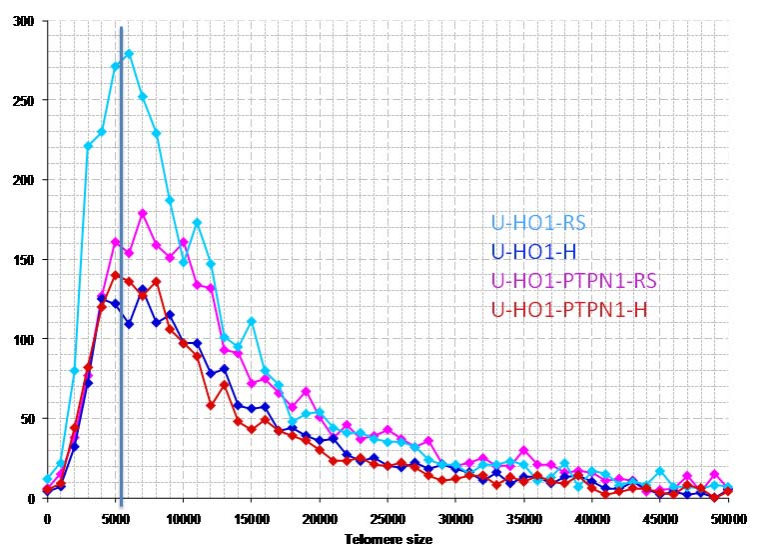
**Telomere distribution according to size in U-HO1 and U-HO1-PTPN1**. Results are based on 3D analysis of at least 30 H-cells and at least 30 bi- or multinucleated RS cells. Grey line at 5000 U demarcates the border between very short telomeres including "t-stumps" and short telomeres. U-HO1 RS-cells (clear blue) show significantly more (p < 0.0001) very short telomeres (0-5000 units) compared to U-HO1-PTPN1 RS-cells (pink). U-HO1-PTPN1 RS-cells have nearly identical numbers of very short telomeres as U-HO1-PTPN1 H-cells consistent with the possibility that PTPN1 expression abolished the formation very short telomeres including "t-stumps" during the transition from H- to RS-cells.

**Figure 6:**
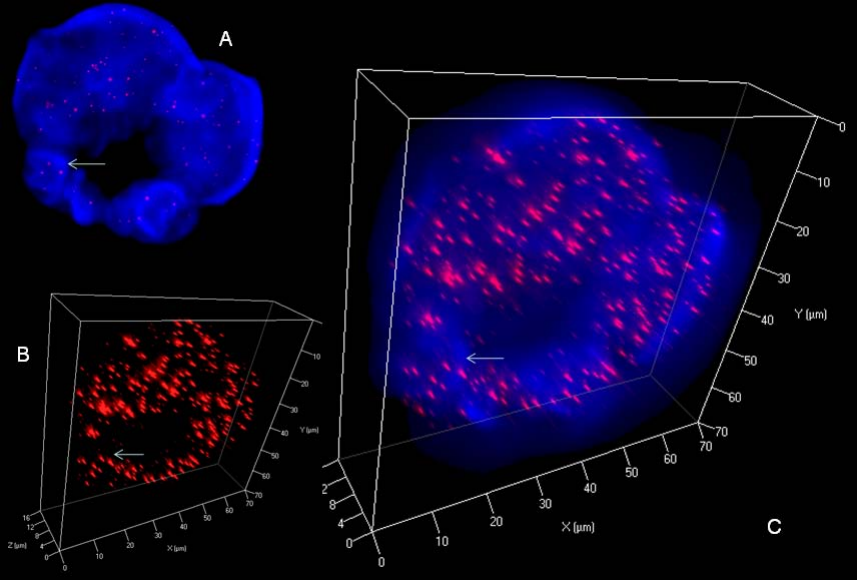
**3D nuclear staining of telomeres and DNA in U-HO1 RS-cells**. A. Representative 2D Z-stack image no. 36 out of 80 shows ring-like multinuclear (blue) RS-cell composed of at least four nuclei of variable size. Arrow identifies a telomere group also shown in B and C. B. 3D reconstruction in surface mode reveals abundant short and very short telomeres (red). C. Combined 3D nuclear staining confirms ring-like nuclear configuration.

**Figure 7:**
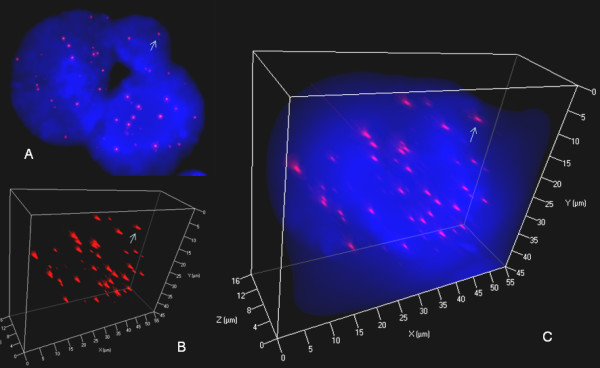
**3D nuclear staining of telomeres and DNA in U-HO1-PTPN1 RS-cells**. A. Representative 2D Z-stack image no. 37 out of 80 shows a tri-nuclear RS-cell composed of two large and one small telomere-poor "ghost" satellite nucleus. Arrow identifies a mid-sized telomere also shown in B and C. B. 3D reconstruction in surface mode reveals relatively few (compared to cellular volume) mid-sized and short telomeres. Very short telomeres including t-stumps are rare. C. Combined 3D nuclear staining confirms 3D telomere dynamics identical to those observed in precursor H-cells (see Figure 4).

Reed-Sternberg cells of both, U-HO1 and U-HO1-PTPN1, showed several common characteristics as previously identified in RS-cells of the HL cell lines HDLM-2, L-428, L-1236 and patient biopsies [4,6]. As expected, U-HO1-RS-cells and U-HO1-PTPN1 RS-cells significantly differed from their mononuclear H-cell precursors by their nuclear volume (p < 0.0001), number of telomeres (p < 0.0001) and increase of telomere aggregates (p < 0.003). Strikingly, RS-cells of U-HO1 differed from U-HO1-PTPN1-RS cells by a highly significant increase in very short telomeres including so-called "t-stumps" (p < 0.0001) (Table 1 Figure 5). RS-cells of U-HO1 often showed numerous very short and short telomeres without loss of mid-sized telomeres and stable number of telomeres/1000 μm^3 ^of nuclear volume, consistent with the hypothesis that these RS-cells were still able to divide further through endoreplication, resulting in giant RS-cells (Figure 6). Indeed, such telomere rich U-HO1-RS cells were regularly identified and telomere-poor or -free "ghost" nuclei were rare. Contrary to the U-HO1 RS-cells, the PTPN1 expressing RS-cells did not show any increase of very short telomeres (Figure 5 and 7) resulting in multinuclear RS-cells with a low number of telomeres/1000 μm^3 ^of nuclear volume when compared to their mononuclear H-precursors (p = 0.0175). In such U-HO1-PTPN1 expressing RS-cells formation of telomere-poor "ghost" nuclei was frequently observed.

## Discussion

The Q-FISH analysis of the 3D nuclear telomere organization in the HL cell line U-HO1 reveals analogous characteristics of multinuclear RS-cells compared to their mononuclear precursor H-cells as recently described in the HD-cell lines HDLM-2, L-428, L-1236 and in diagnostic biopsies of EBV-negative and LMP1-expressing classical HL [4,6]. RS-cells of U-HO1 have a 3-4 times higher nuclear volume, a highly significant increase of telomere aggregates, and a substantial increase of very short telomeres, including so-called "t-stumps", a hallmark of cancer cells [16].

But contrary to the RS-cells of HDLM-2, L-428 and L-1236, which show a significant loss of telomeres/1000 μm^3 ^of nuclear volume compared to their mononuclear precursor H-cells, the RS-cells of U-HO1 show still the same number of telomeres/1000 μm^3 ^of nuclear volume as their mononuclear H-cell precursors, consistent not only with a high telomerase activity but also with a still functioning shelterin complex allowing correct telomere elongation [17]. Indeed, U-HO1 is far less genetically aberrant than other HL cell lines [18,19] but has about a six-fold copy number gain of *REL *and a strong corresponding nuclear *c-REL *protein localization, indicating a high nuclear NF-κB transcriptional activity [7] which is required for proliferation and survival of H- and RS-cells [20]. Phosphorylated STAT5, whose expression and activation is controlled by NF-κB, and which is also required for HL lymphomagenesis [12], is identified in the cytoplasm of H- and RS-cells in U-HO1 [[8], present study].

We hypothesize that the telomere dynamics identified in U-HO1 reflect accumulation of phosphorylated STAT5 resulting in permanent Akt/PKB (protein kinase B) activation, for the following reasons: i) in HL accumulation of phosphorylated STAT5 in H- and RS-is frequent and possibly due to the expression of a mutated, non functional SOCS-1 gene (suppressor of cytokine signalling 1 gene) since single cell analysis of microdissected H- and RS-cells from patient biopsies showed a significant correlation between high nuclear phosphorylated STAT5 expression and SOCS-1 gene mutations [21], ii) cytoplasmic accumulation of phosphorylated STAT5 is identified in chronic and acute myeloid leukemias and constitutive STAT5 activation promotes leukemogenesis through cytoplasmic complex formation with PI3-K/Gab2 resulting in Akt/PKB activation [22], iii) activation of Akt/PKB is needed for G_2_/M transition and may override a G_2 _arrest induced by DNA damage [23]. This abrogation of the mitotic spindle cell-cycle checkpoint leads indeed to polyploidization in vascular smooth muscle cells transfected with Akt1 [24] and might explain why some RS-cells of U-HO1 progress to >8N DNA content through endoreplication while still conserving a stable number of telomeres/1000 μm^3^.

PTPN1 may exert tumor suppressing or tumor promoting effects depending on the substrate involved and the cellular context [25,26]. In the context of U-HO1, stable expression of PTPN1 in the same cell line had highly significant effects on slowing down the proliferation, on induction of apoptosis, and on increasing the formation of RS-cells. These RS-cells had a significant loss of the number of telomeres/1000 μm^3 ^of nuclear volume but no increase in the number of very short telomeres including "t-stumps" compared to the mononuclear H-precursors. Thus, stable PTPN1 expression was associated with dissociation of the reported 3D telomere dynamics during RS-cell formation, resulting in numerous RS-cells with a precursor H-cell 3D telomere profile (Figure 5 and 7).

Since PTPN1 specifically deactivates phosphorylated STAT5A and STAT5B [9] and stable expression of siRNA targeting STAT5 slows down the proliferation of L-428 and L-1236 cells by 200-300% [12], we hypothesize PTPN1 induced dephosphorylation of STAT5 in U-HO1-PTPN1 cells to be the mechanism slowing down the proliferation. In particular, the consecutive lack of PI3-K/Gab2 and Akt/PKB activation needed for G_2_/M transition might alleviate anti-apoptotic potential, result in a G_2 _arrest induced by DNA damage [23] and accumulation of end-stage RS-cells. The lack of any increase of very short telomeres in U-HO1-PTPN1 RS-cells, contrary to the significant increase marking the transition from H- to RS-cells in HDLM-2, L-1236, U-HO1 and, most importantly, in patient biopsies, is consistent with the induction of apoptosis by PTPN1 expression since induction of apoptosis does not change the abundance of "t-stumps" in cancer cells [16].

## Conclusions

Our Q-FISH analysis of the 3D telomere dynamics in the HL cell lines U-HO1 and U-HO1-PTPN1 reveals that stable PTPN1 expression is associated with i) low proliferation rate, ii) increased number of RS-cells, iii) induction of apoptosis, and iv) prevention of the additional formation of very short telomeres and "t-stumps", but not interfering with other 3D telomere dynamics associated with RS-cell formation. Whether PTPN1 induced dephosphorylation of cytoplasmic STAT5 is at the origin of these profound changes has to be addressed by further experimental studies.

## Methods

### Cell lines

The cell lines U-HO1, U-HO1-PTPN1 and U-HO1-mock were grown in suspension in 25 cm^2 ^culture flasks in Iscove/RPMI-1640 medium (4:1) supplemented with 10% fetal calf serum, L-glutamine (2 mM), penicillin (100 U/ml) and streptomycin (100 U/ml) in 5% CO_2 _in a humidified atmosphere at 37^o ^C. U-HO1 and U-HO1-mock were passed into new flasks containing fresh culture media twice weekly, U-HO1-PTPN1 once weekly, due to much slower growth.

### Generation of U-HO1-PTPN1

Plasmids were transfected into U-HO1 cells using the Nucleofector device (Amaxa, Cologne, Germany). 2 × 10^6 ^cells and 4 μg expression vector were used for each plasmid nucleofection (Nucleofector Kit V, program X-01). pcDNA4/TO vector was obtained from Invitrogen (Invitrogen, San Diego, CA) and used as a control mock-vector. The *PTPN1 *coding sequence (NM 003745, nucleotide 155 to 790) was amplified by PCR on cDNA from human tonsil and inserted into pcDNA4/TO vector under control of CMV promoter. The plasmids were purified using Endofree Plasmid Purification MaxiKit (Qiagen, Hilden, Germany). *PTPN1 *sequence was checked by sequencing (MWG Biotech, Ebersberg, Germany). Cells were harvested 24 h and 48 h post transfection or after at least 4 weeks under zeocin selection for stable transfection. Success of stable transfection was proved by immunoblotting against PTPN1.

### TRAP assay

TRAP (Telomeric Repeat Amplification Protocol) was performed using the TRAPeze Telomerase detection Kit (S7700, Chemicon, Temecula, CA, USA). 100 ng of protein extract were subject to the TRAPeze procdure with and without heat inactivation of telomerase (ΔT). Pictures were shown as the negative of Ethidium bromide coloration of a 10% PAGE (TBE 0.5X).

### Apoptosis

Apoptosis was assessed by flow cytometry according to the standard procedure of Nicoletti et al. [27].

### Immunoblotting

For Western blotting 20 μg of protein were charged per lane. The following primary antibodies were used: PTPN1 (murine clone AE4-2J) (Merck, Darmstadt, Germany), β-actin (murine clone AC-74), (Sigma, Deisenhofen, Germany). For detection peroxidase conjugated goat anti-rabbit IgG or goat anti-mouse IgG, (Peribo Science, Bonn, Germany) was used. Signals were visualized using the SuperSignal West Dura extended-duration substrate (Perbio Science, Aalst, Belgium).

### Immunocytology

Cytocentrifuge preparations of U-HO1 and and U-HO1-PTPN1 cells were fixed in acetone (5 min), air dried, and stained with MayGrünwald/Giemsa (MGG) according to standard conditions. For the detection of phospho-STAT5, fresh cells of U-HO1 and U-HO1-PTPN1 were collected, centrifuged, fixed in ethanol and embed in paraffin. The specimen were cut in 5 μm thick sectiones and further treated as usual. The primary monoclonal antibody against phosphorylated STAT5 (Cell Signaling Technology, Beverly, MA) was diluted of 1:50 and used according to manufactorers instructions. For detection the EnVision detection system (Dako, Copenhagen, Denmark) was used and 3-Amino-9-ethylcarbazole served as the substrate.

### Telomere Q-FISH

U-HO1 and U-HO1-PTPN1 cells were collected (200 × *g *for 10 min) and resuspended in PBS containing 3.7% formaldehyde (Fluka) and incubated for 20 min. Thereafter, the telomere Q-FISH protocol was performed [5,28] by using Cy3-labelled PNA probes (DAKO). Imaging of interphases after telomere FISH was performed by using Zeiss AxioImager Z1 with a cooled AxioCam HR B & W, DAPI, Cy3 filters in combination with a Planapo 63 ×/1.4 oil objective lens. Images were acquired by using AXIOVISION 4.6 (Zeiss) in multichannel mode followed by constraint iterative deconvolution as specified below.

### 3D Image Acquisition

At least 30 H-cell interphase nuclei and at least 30 RS-cell interphase polykaria were analyzed for both cell lines. AXIOVISION 4.6 with deconvolution module and rendering module were used. For every fluorochrome, the 3D image consists of a stack of 80 images with a sampling distance of 200 nm along the *z *and 107 nm in the *xy *direction. The constrained iterative algorithm option was used for deconvolution [29].

### 3D Image Analysis for Telomeres

Telomere measurements were done with TeloView TM [30]. By choosing a simple threshold for the telomeres, a binary image is found. Based on that, the center of gravity of intensities is calculated for every object resulting in a set of coordinates (*x, y, z*) denoted by crosses on the screen. The integrated intensity of each telomere is calculated because it is proportional to the telomere length [31].

### Telomere aggregates

Telomere aggregates are defined as clusters of telomeres that are found in close association and cannot be further resolved as separate entities at an optical resolution limit of 200 nm [32].

### Telomere length

Telomeres with a relative fluorescent intensity (y-axis) ranging from 0-5000 units are classified as very short, with an intensity ranging from 5,000-15,000 units as short, with an intensity ranging from 15,000-30,000 units as mid-sized, and with an intensity >30,000 units as large. The cut-off of 5,000 units is arbitrary and based on our 5 years' experience. Telomeres with an intensity ≪ 5'000 are still easily identifiable on the Teloview-Program, but barely detectable on 3D reconstitution images.

### Telomere volume

Total telomere volume is the sum of all very short, short, mid-sized and large telomeres and aggregates within one H- or RS-cell.

### Nuclear volume

The Nuclear volume is calculated according to the 3D nuclear 4',6-diamidino-2-phenylindoline staining as described earlier [33].

### Statistical analysis

For both cell lines, normally distributed parameters are compared between the two types of cells using nested or two-way analysis of variance. Multiple comparisons using the least-square means tests followed in which interaction effects between two factors were found to be significant. Other parameters that were not normally distributed were compared using a non-parametric Wilcoxon rank sum test. Significance levels were set at P = 0.05. Analyses were perfirmed using SAS v9.1 programs.

## Authors' contributions

HK, SB and SM conceived the study and established its initial design. HK, SB, SW, ZL and BL carried out experimental work, HK, DL, PM and SM performed experimental data analysis, HK prepared the manuscript and SB, PM and SM performed critical revisions. All authors have read and approved the final manuscript.
